# The American Dental Association

**Published:** 1880-07

**Authors:** 


THE AMERICAN DENTAL ASSOCIATION will hold its twentieth annual session August 3d, 4th, 5th and 6th, 1880, at the Hall of the Mass. Institute of Technology, Boston, Mass. Hotel accommodations at the "Brunswick," Boylston, corner Claredon streets, opposite the Institute. Rates $3.00 per day, reduced from $4.50. This hotel is new and first class in every appointment. The Hotel and Hall are in near proximity to the business part of the city, yet free from the noise and turmoil incident to the business streets. The committee of arrangements feel sure they will be able to provide opportunities to make the stay of members pleasant and the meetings of the association profitable. THOMAS FILLEBROWN,
Chairman 1st Div. Ex. Com.

EAST TENNESSEE DENTAL ASSOCIATION meets 4th of August at Chattanooga.

THE NEW JERSEY STATE DENTAL SOCIETY meets 21st inst., (continues three days,) which is one day later than given in our last issue. The Examining Board meets on the 20th, all at Long Branch.

